# A review of 91 canine and feline red‐bellied black snake (*Pseudechis porphyriacus*) envenomation cases and lessons for improved management

**DOI:** 10.1111/avj.13159

**Published:** 2022-03-23

**Authors:** MK Wun, AM Padula, RM Greer, EM Leister

**Affiliations:** ^1^ Veterinary Specialist Services 1‐15 Lexington Rd Underwood Queensland 4119 Australia; ^2^ William R. Pritchard Veterinary Medical Teaching Hospital, School of Veterinary Medicine University of California Davis California 95616 USA; ^3^ Australian Venom Research Unit, Department of Pharmacology and Faculty of Medicine, Dentistry and Health Sciences University of Melbourne Melbourne Victoria 3010 Australia; ^4^ Padula Serums Bairnsdale Victoria 3875 Australia; ^5^ Torus Research Brisbane Queensland Australia; ^6^ Faculty of Medicine The University of Queensland St Lucia Queensland 4072 Australia; ^7^ Pet Intensive Care Unit 1‐15 Lexington Rd Underwood Queensland 4119 Australia

**Keywords:** acute kidney injury, dog, *Pseudechis porphyriacus*, red‐bellied black snake, snake envenomation, veterinary

## Abstract

**Introduction:**

Most cases of red‐bellied black snake (RBBS) envenomation in dogs respond favourably to treatment comprising of tiger‐brown snake antivenom (TBAV), intravenous fluid therapy, analgesia and, if indicated, mechanical ventilation and/or blood transfusion. However, there remains a subset of patients who develop fatal complications despite intensive treatment and risk factors for these occurring remain unknown. Here we present a retrospective cross‐sectional survey of 91 canine and feline RBBS envenomation cases.

**Methods:**

Cases seen between June 2010 and June 2020 were retrieved from the databases of seven practices in South East and coastal Queensland. From the canine case population, logistic regression analysis was performed to assess the impact of potential risk factors at presentation on the likelihood of death. A final multivariable model was developed using a manual backwards elimination approach based on overall likelihood ratio tests and Wald chi‐square P‐values for each variable. Where model convergence failed due to quasi‐complete separation, Firth's penalised maximum likelihood method was implemented. Such separation may occur when an outcome is completely predicted by an explanatory variable in one group.

**Results:**

Of the 88 canine cases, 7 died (8.0%), all after prognosis‐based euthanasia. Of the three feline cases, one died after unsuccessful resuscitation following cardiopulmonary arrest. Compared to survivors, dogs that died were older, exhibited pigmenturia, received antivenom later and had a higher total plasma protein (TPP), activated clotting time (ACT) and lower packed cell volume (PCV) at presentation.

The red‐bellied black snake (RBBS; *Pseudechis porphyriacus*) is one of six species of the *Pseuedechis* genus found in Australia and the only one that appears consistently uniformly black when seen from above.^1^ Its geographical distribution extends along the eastern coast extending from Far North Queensland to Victoria and also in South Australia between Adelaide and the lower Murray River.^1^ Despite its shy and non‐aggressive nature, the envenomation of dogs is relatively common in endemic areas.^2^, ^3^, ^4^ RBBS venom contains strong haemolysins, mild neurotoxins, myotoxins, cytotoxins and anti‐ and pro‐coagulants,^5^, ^6^, ^7^, ^8^ which results in the canine toxidrome of haemolysis, myopathy, pigmenturia, soft tissue swelling at the bite site and a coagulopathy that is usually mild and subclinical.^2^, ^3^ Paresis, respiratory compromise, collapse episodes, hypersalivation, vomiting, diarrhea, facial palsy and haemoptysis may also occur.^2^, ^3^ Reported clinical pathology findings include moderate to severely increased creatine kinase (CK), hyperbilirubinaemia, elevated alanine aminotransferase (ALT), alkaline phosphatase (ALP) and aspartate aminotransferase (AST), prolonged activated clotting time (ACT) and spherocytosis.^2^, ^3^ Haemolytic anaemia is usually only present in severe cases.^2^, ^3^, ^9^

Most cases respond favourably to intravenous fluid therapy (IVFT), analgesia and combined tiger and brown snake antivenom (TBAV),^3^ due to the immunological cross‐reactivity of tiger and black snake venom components.^10^ One TBAV vial containing 3000–4000 units of tiger snake antivenom is generally sufficient and binds all free circulating venom, although multiple vials are often given in cases with severe and/or ongoing haemolysis.^2^, ^3^, ^9^ A monovalent black snake antivenom registered for use in humans is available,^11^ though is not commonly used by veterinarians due to its high cost and a comparatively large volume of administration. In patients with severe anaemia or neurotoxicity, blood transfusion or mechanical ventilation, respectively, may be required.^2^, ^3^, ^9^, ^12^

Although most dogs recover, some without the benefit of antivenom, there are isolated reports of patients who develop fatal, anuric acute kidney injury (AKI) despite receiving TBAV and intensive supportive treatment.^3^, ^13^ Necropsy and histopathological findings in one dog included generalised icterus, tri‐cavitary serosanguinous effusion, pulmonary congestion and haemorrhage and enlarged and dark red‐black kidneys with pigment casts and sloughed epithelial cells present within the tubules.^13^ These findings are suggestive of myohaemoglobinuric AKI due to continued haemolysis and rhabdomyolysis rather than direct venom nephrotoxicity.^13^ Due to the paucity of reported cases, risk factors for death and early therapeutic strategies to prevent or mitigate AKI occurrence in dogs and cats are unclear. To amend this knowledge gap, here we report an analysis of 91 canine and feline RBBS envenomation cases with the aim to identify risk factors for mortality. An ancillary aim was to further describe the clinical presentations, laboratory findings, treatments and outcomes of RBBS envenomation in an expanded cohort of animals, to complement and consolidate earlier work.^2^, ^3^

## Materials and methods

### Case selection

Clinical records from June 2010 to June 2020 containing the words ‘RBBS’ or ‘black snake’ were retrieved from the databases of seven practices in South East and coastal Queensland; Veterinary Specialist Services (VSS) Underwood, Jindalee and Carrara (ezyVet®; ezyVet, New Zealand), Animal Emergency Service (AES), Underwood, Jindalee and Carrara (RxWorks®; Covetrus, Australia) and Animal Referral Hospital (ARH), Sinnamon Park (RxWorks; Covetrus). VSS and AES operate within the same premises, managing day‐time and after‐hours cases, respectively. All cases with a presumptive or definitive diagnosis of RBBS envenomation were enrolled and relevant data from their records extracted. A presumptive diagnosis was made based on a combination of owner history of observed animal‐snake exposure, positive snake identification by scale counting (17 mid‐body scales) and appearance (1–2 m long, black dorsum, red ventrum) by the treating veterinarian or a herpetologist and suggestive clinical signs (haemolysis, myopathy, pigmenturia, bite site swelling and/or coagulopathy). A definitive diagnosis was made based on a combination of suggestive clinical signs and a positive urine snake venom detection kit (SVDK; Seqirus, Parkville, Victoria, Australia) result for black snake, or retrospective detection of RBBS venom antigen in frozen serum or urine using a RBBS‐specific sandwich enzyme‐linked immunosorbent assay (ELISA) which is highly specific for RBBS venom with little cross‐reactivity with tiger snake (*Notechis scutatus*) (<10%) and brown snake (*Pseudonaja textilis*) (<1%) venom.^2^ To maximize statistical power, 17 previously published cases were included.^3^ Cases referred to VSS, AES or ARH for management after being previously diagnosed or treated for RBBS envenomation elsewhere were excluded. Cases that were discharged were not followed up but were assumed to have survived.

### Seasonal distribution, demographics, clinical presentation and clinical pathology

For each case, signalment, the month of presentation, body weight, clinical signs, time from the bite to antivenom administration and packed cell volume (PCV), total plasma protein (TPP), coagulation studies, venous blood gas and serum biochemistry results at presentation, if performed, were extracted. The season of presentation was classified as summer (December–February), autumn (March–May), winter (June–August) and spring (September–November). Serum biochemistry results during hospitalisation, where available, were also evaluated for evidence of AKI and elevated ALT and ALP. Gait and respiratory scores were assigned using a modified tick scoring system as previously described (Table 1).^14^ Time from bite to antivenom was estimated from the owner history and classified as <6, 6–12, 12–24 or >24 h. Haemolysis was defined as pink discolouration of plasma following centrifugation and pigmenturia as gross urine discolouration that did not resolve following centrifugation. TPP was estimated using refractometry. ACT was performed using commercial tubes (MAX‐ACT® or C‐ACT®; Helena Laboratories, Mt. Waverly, Victoria, Australia) and a whole blood coagulation monitor (HEMOCHRON® 401; International Technidyne Corporation, Hallam, Victoria, USA) and the standard normal deviate (SND) calculated from previously defined reference intervals.^3^ A prolonged ACT was defined as a SND >2. Prothrombin time (PT) and activated partial thromboplastin times (aPTT) were measured using a VETSCAN® VSpro Coagulation Analyzer (Zoetis, Parkville, Victoria, USA) (VSS and AES) or Coag Dx Analyser (IDEXX Laboratories, East Brisbane, Queensland, Australia) (ARH). In‐house venous blood gas and electrolytes analysis from 2016 onwards were performed using a GEM Premier® 5000 (Instrumentation Laboratory, USA), with a GEM Premier® 4000 (Instrumentation Laboratory) used prior to this. Serum biochemical analysis was performed using a Catalyst One® (IDEXX Laboratories) machine or at a reference laboratory (QML Pathology [Murarrie, Queensland, Australia]).

**Table 1:** Snakebite severity scoring system^11^

Clinical sign	Score	Description
Gait	0	No clinical signs
	1	Mild paresis, able to ambulate
	2	Able to stand/sit unaided but cannot walk
	3	Unable to stand but can maintain sternal recumbency
	4	Unable to maintain sternal recumbency
Respiratory	1	No compromise
	2	Mild increase in effort and/or respiratory rate
	3	Moderate, respiratory rate < 16 or > 40, minimal excursions, abdominal component
	4	Dyspnoea, cyanosis, unsustainable respiratory pattern, respiratory arrest, imminent death

### Complications

Complications including antivenom reaction, arrhythmia, AKI, systemic inflammatory response syndrome (SIRS), multiple organ dysfunction syndrome (MODS), acute respiratory distress syndrome (ARDS) and haemorrhage were recorded. AKI was classified as per International Renal Interest Society (IRIS) guidelines into grades based on serum creatinine concentrations and subgrades based on urine output (non‐oliguric [NO] or oligo‐anuric [O]).^15^ A diagnosis of SIRS was made in dogs exhibiting at least 2 of the following changes; rectal temperature < 38.1°C or > 39.2°C, heart rate > 120 beats per minute, respiratory rate > 20 breaths per minute and a white blood cell count x10^9^/L; % bands < 6 or >16; >3%.^16^ MODS was defined as ‘the development of potentially reversible physiologic derangement involving two or more organ systems not involved in the disorder that resulted in ICU admission and arising in the wake of a potentially life‐threatening physiologic insult’.^17^ Acute respiratory distress syndrome was diagnosed in cases with respiratory symptoms developing during the course of hospitalisation, radiographic evidence of bilateral lung opacities not fully explained by effusions, lobar/lung collapse or nodes, respiratory failure not fully explained by cardiac failure or fluid overload and a PaO_2_/FiO_2_ ratio < 300 mmHg with PEEP ≥ 5 cm H_2_O.^18^

### Outcomes

The primary outcome was mortality status (death or survival) and the secondary outcome was days in hospital. The total duration (days) of hospitalisation was recorded and cases were categorised as having survived or died. Cases that were discharged were not followed‐up but were assumed to have survived.

### Statistical analysis

Canine case data was analysed using SPSS® Statistics (IBM®, New York, NY, USA) and SAS 9.4 (SAS Institute, Cary, NC, USA). The individual instance of RBBS envenomation (i.e., each case) was the unit of analysis. Continuous data were assessed for normality using graphical methods. Summary statistics are reported as n, mean (SD, standard deviation) for normally distributed continuous data and n, median (range) for non‐normally distributed continuous data, where n is the number of cases where the variable of interest was available to be extracted from the medical record. PCV, TPP, ACT and iCa were considered continuous variables throughout. Categorical data were reported as n/N (%), where n is the number of affected animals and N the total number of animals. Associations between potential risk factors and mortality status were assessed using Pearson Chi‐Square or Fisher's Exact Test for categorical variables and Mann–Whitney U test for continuous variables. Variables with biological plausibility, an adequate sample size and a P‐value ≤ 0.3 were selected for initial multivariable logistic regression analysis with death as the dependant variable. A final multivariable model was developed using a manual backwards elimination approach based on overall likelihood ratio tests and Wald chi‐square P‐values for each variable. Significance was determined by a P‐value ≤ 0.05. Further logistic regression analyses were also performed on subsets of cases where complete data for all potential risk factors identified were available. Where model convergence failed due to quasi‐complete separation, Firth's penalised maximum likelihood method^19^, ^20^ was implemented using SAS PROC LOGISTIC with the FIRTH option. Such separation may occur when an outcome is completely predicted by an explanatory variable in one group, for example, when a risk factor is present in all subjects in one group. Due to small case numbers, statistical analysis was not performed for feline cases and results are described within the text instead.

## Results

### Number of cases

A total of 91 cases meeting our inclusion criteria were found. Of these, 19 cases were presumptively diagnosed, while the remaining 72 were definitively diagnosed based on a positive urine SVDK (55 cases), positive ELISA result (8 cases) or positive SVDK and ELISA (9 cases). The 91 cases that met the inclusion criteria occurred in 89 animals; 87 animals presented with RBBS envenomation on a single occasion during the study period, while 2 animals presented on two occasions.

### Signalment

There were 88 dogs and 3 cats. The dogs comprised of 21 Staffordshire Bull Terriers and 4 Staffordshire Bull Terrier crossbreds, 10 Jack Russel Terriers, 3 Labrador Retrievers and 2 Labrador Retriever crossbreds, 2 Australian Cattle Dogs and 2 Australian Cattle Dog crossbreds, 2 American Staffordshire Terriers and 1 American Staffordshire Terrier crossbred, 2 Boxers and 1 Boxer crossbred, 1 Bull Arab and 2 Bull Arab crossbreds, 1 Kelpie and 2 Kelpie crossbreds, 1 Toy Poodle and 1 Toy Poodle crossbred, 1 Fox Terrier and 1 Fox Terrier crossbred, 2 Great Dane crossbreds, 1 Miniature Fox Terrier and 1 Miniature Fox Terrier crossbred, 1 Rhodesian Ridgeback and 1 Rhodesian Ridgeback crossbred, 2 Weimaraners and 1 each of a wide variety of other breeds and crossbreds. Age, gender and bodyweight from these cases are presented in Table 2; only age was associated with mortality status (P = 0.007). The cats comprised 1 male neutered Burmese, 1 male neutered Ragdoll and 1 female spayed Domestic Shorthair. Age and bodyweights ranged from 30–71 months and 4–6 kg, respectively.

**Table 2:** Summary statistics for signalment, seasonal distribution, clinical presentation and antivenom treatment for 88 canine RBBS envenomation cases and associations with mortality status

Variable and categories	All cases (n = 88)	Survivors (n = 81)	Non‐survivors (n = 7)	P‐value
Age (M)	67.5 (5–161)	59 (5–143)	107 (65–161)	0.01
Sex				0.70
Male neutered	35 (39.8)	31 (38.3)	4 (57.1)	
Male entire	7 (8.0)	7 (8.6)	0 (0)	
Female spayed	35 (39.8)	33 (40.7)	2 (28.6)	
Female entire	11 (12.5)	10 (12.3)	1 (14.3)	
Weight (kg)	21.5 (4.0–67.0)	23.0 (4.0–67.0)	21.0 (7.4–40.0)	0.79
Season				0.94
Summer	32 (36.4)	30 (37.0)	2 (28.6)	
Autumn	26 (29.5)	24 (29.6)	2 (28.6)	
Winter	8 (9.1)	7 (8.6)	1 (14.3)	
Spring	22 (25.0)	20 (24.7)	2 (28.6)	
Bite site oedema				0.70
No	40 (45.5)	36 (44.4)	4 (57.1)	
Yes	48 (54.4)	45 (55.6)	3 (42.9)	
Pigmenturia				0.002
No	49 (55.7)	49 (60.5)	0 (0)	
Yes	39 (44.3)	32 (39.5)	7 (100)	
Gait score				0.95
0	61 (69.3)	56 (69.1)	5 (71.4)	
1	20 (22.7)	18 (22.2)	2 (28.6)	
2	1 (1.1)	1 (1.2)	0 (0)	
3	4 (4.5)	4 (4.9)	0 (0)	
4	2 (2.3)	2 (2.5)	0 (0)	
Hypersalivation				0.19
No	61 (69.3)	58 (71.6)	3 (42.6)	
Yes	27 (30.7)	23 (28.4)	4 (57.1)	
Vomiting				1.00
No	68 (77.3)	62 (76.5)	6 (85.7)	
Yes	20 (22.7)	19 (23.5)	1 (14.3)	
Respiratory score				0.27
0	69 (78.4)	64 (79.0)	5 (71.4)	
1	13 (14.8)	12 (14.8)	1 (14.3)	
2	1 (1.1)	1 (1.2)	0 (0)	
3	3 (3.4)	3 (3.7)	0 (0)	
4	2 (2.3)	1 (1.2)	1 (14.3)	
Cranial nerve abnormalities				1.00
No	81 (92.0)	74 (91.4)	7 (100)	
Yes	7 (8.0)	7 (8.6)	0 (0)	
Diarrhea				0.45
No	81 (92.0)	75 (92.6)	6 (85.7)	
Yes	7 (8.0)	6 (7.4)	1 (14.3)	
Collapse episode				1.00
No	84 (95.5)	77 (95.1)	7 (100)	
Yes	4 (4.5)	4 (4.9)	0 (0)	
Facial palsy				0.22
No	85 (96.6)	79 (97.5)	6 (85.7)	
Yes	3 (3.4)	2 (2.5)	1 (14.3)	
Haemorrhage				0.08
No	87 (98.9)	81 (100)	6 (85.7)	
Yes	1 (1.1)	0 (0)	1 (14.3)	
Vials of AV				0.97
1	65 (73.9)	60 (74.1)	5 (71.4)	
2	21 (23.9)	19 (23.5)	2 (28.6)	
3	1 (1.1)	1 (1.2)	0 (0)	
4	1 (1.1)	1 (1.2)	0 (0)	
Antivenom				1.00
Summerlands	65 (73.9)	60 (74.1)	5 (71.4)	
Padula	23 (26.1)	21 (25.9)	2 (28.6)	
Bite to antivenom time	n = 82	n = 76	n = 6	0.01
<6 h	50 (61.0)	50 (65.8)	0 (0)	
6–12 h	14 (17.1)	12 (15.8)	2 (33.3)	
12–24 h	8 (9.8)	7 (9.2)	1 (16.7)	
>24 h	10 (12.2)	7 (9.2)	3 (50.0)	

Categorical variables are reported as n (%). Continuous variables are reported as median (range).

### Seasonal distribution

**Figure 1:**
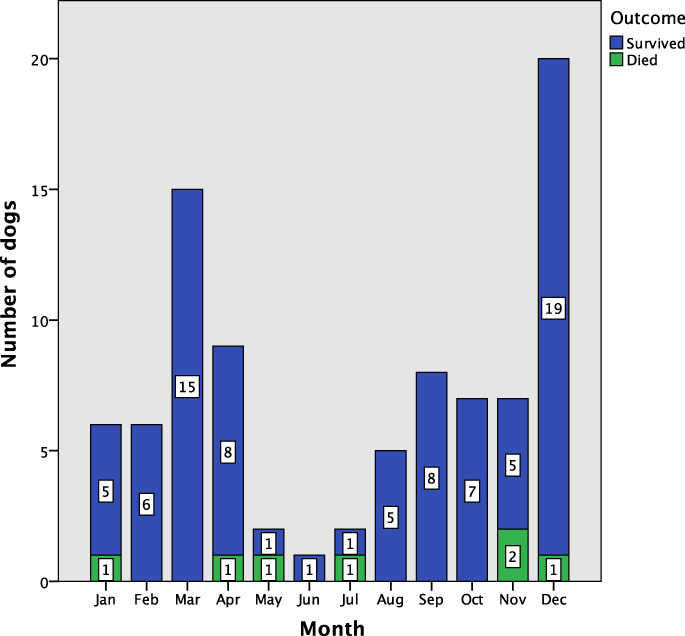
Number of canine RBBS envenomation cases by month and outcome during the study period (total = 88). The *y*‐axis is the number of dogs, while the month of presentation is given along the *x*‐axis.

Canine cases occurred all year, with numbers peaking in December (start of summer) and declining from May–June (late‐autumn to winter) (Table 2 and Figure 1). Most cases occurred in summer, autumn and spring, with the fewest occurring in winter. There was no effect of season on mortality status (P = 0.941). The feline cases occurred in spring, summer and autumn.

### Clinical presentations

The most common clinical signs in dogs were localised bite site oedema, followed by pigmenturia, elevated gait score (≥1), hypersalivation, elevated respiratory score (≥1), vomiting, cranial nerve abnormalities (dilated pupils and/or absent pupillary light, palpebral and/or gag reflex), diarrhoea, collapse episode, facial palsy associated with the bite site and haemorrhage (hyphaema, haemothorax, haemoptysis and pulmonary haemorrhage) (Table 2). Eleven/88 dogs (12.5%) exhibited no clinical signs at presentation. Of the clinical signs, only pigmenturia was associated with mortality status, present in 32/81 (38.5%) of survivors and 7/7 (100%) in dogs that died (P = 0.002). Eighteen of 61 (29.5%) dogs with a bite to antivenom time of <12 h developed pigmenturia, compared with 18/18 (100%) of dogs where the bite to antivenom time was ≥12 h (P < 0.001). Clinical signs seen in cats included bite site oedema (1/3), pigmenturia (1/3), elevated gait score (1/3), elevated respiratory score (1/3), cranial nerve abnormalities (1/3) and collapse episode (1/3). Bite to antivenom times were < 6 h (1/3), 6–12 h (1/3) and >24 h (1/3).

### Clinical pathology

Clinical pathology for canine cases at presentation is presented in Table 3. Envenomed dogs, variable, showed haemolysed serum, anaemia, elevated TPP, prolonged ACT, PT and aPTT, acidaemia, hypocalcaemia (based on internal carotid artery [iCa]), hypokalaemia, hyperlactataemia and elevated CK, bilirubin, ALT and ALP, relative to the reference interval. Of this, 11 out of 62 (17.7%) dogs had non‐haemolysed serum and ACT within the reference interval. PCV, ACT and iCa at presentation were associated with mortality status (P ≤ 0.04). Clinical pathology abnormalities seen in cats included haemolysis (1/3), prolonged ACT (1/3), acidaemia (1/2), hyperlactataemia (1/1) and elevated ALT (2/2).

**Table 3:** Summary statistics for clinical pathology variables at presentation for 88 canine RBBS envenomation cases and associations with mortality status

Variable and categories	All cases	Reference interval	Survivors	Non‐survivors	P value
PCV (%)	n = 85; 47.84 (14.07)	35–57	n = 78; 49.35 (13.39)	n = 7; 31.00 (10.39)	0.001
TPP (g/L)	n = 85; 70.71 (11.02)	60–75	n = 78; 69.68 (9.36)	n = 7; 82.14 (20.26)	0.11
Haemolysis					0.92
No	28 (31.8)		28 (34.6)	0 (0)	
Yes	60 (68.2)		53 (65.4)	7 (100)	
ACT (SNDs)	**n = 62; 5.36 (−1.83–20.52)**		n = 57; 4.84 (−1.83–13.20)	n = 5; 8.63 (5.23–20.52)	0.04
PT (s)	n = 13; 16.5 (11.0–26.0)	14–20	n = 10; 17.45 (13.4–26.0)	n = 3; 15.70 (11.0–16.9)	0.37
aPTT (s)	n = 13; 116.0 (81.0–200.0)	94–123	n = 10; 121.9 (81.0–200.0)	n = 3; 89.1 (88.1–100.0)	0.11
Blood pH	n = 55; 7.37 (7.07–7.48)	7.35–7.45	n = 50; 7.37 (7.07–7.48)	n = 5; 7.40 (7.32–7.44)	0.49
K+ (mmol/L)	n = 56; 3.7 (2.7–4.7)	3.4–4.9	n = 51; 3.7 (2.7–4.7)	n = 5; 4.1 (2.8–4.5)	0.21
iCa (mmol/L)	n = 55; 1.28 (0.08)	1.12–1.40	n = 50; 1.29 (0.07)	n = 5; 1.18 (0.09)	0.03
Lactate (mmol/L)	n = 55; 2.0 (0.6–14.7)	0.5–2.0	n = 50; 1.95 (0.6–14.7)	n = 5; 2.6 (1.0–4.6)	0.62
HCO3‐ (mmol/L)	n = 55; 22.6 (3.25)	15–23	n = 50; 22.59 (3.38)	n = 5; 22.26 (1.69)	0.66
Base excess (mmol/L)	**n = 54; −2.65 (−14.7–5.4)**	−5‐2	n = 49; −2.50 (−14.7–5.4)	n = 5; −3.80 (−4.3–0)	0.98
CK (U/L)	**n = 14; 1326 (126–12,925)**	0–400	n = 14; 1326 (126–12,925)	n = 0	
Bilirubin (μmol/L)	**n = 14; 17.5 (1–477)**	0–10	n = 13; 8.0 (1–292)	n = 1; 477	0.14
ALT (U/L)	n = 18; 74.5 (26–456)	0–80	n = 17; 72.0 (26–456)	n = 1; 202	0.33
ALP (U/L)	n = 16; 14.0 (5–926)	1–120	n = 16; 14.0 (5–926)	n = 0	

Categorical variables are reported as n (%). Continuous variables are reported as n; mean (SD) for normally distributed continuous data and n; median (range) for non‐normally distributed continuous data.

Results outside the reference interval are given in **bold** text.

### Treatment

All canine cases received 1–4 vials of TBAV; most received Tiger/Multi‐brown Snake Antivenom (Summerland Serums, Alstonville, NSW, Australia) containing 3000 units of tiger snake antivenom, whilst the remainder received Tiger‐Brown Snake Antivenom (Padula Serums, Bairnsdale, Victoria, Australia) containing 4000 units of tiger snake antivenom (Table 2). Brand and number of vials of TBAV administered were not associated with mortality status (P = 1.0 and 0.97, respectively). In this 40 out of 88 (45.4%) cases received premedication prior to antivenom administration (various combinations of prednisolone sodium succinate 2 mg/kg IV, chlorpheniramine 0.3–1.4 mg/kg IV, IM or SQ and/or adrenaline 0.02–0.04 mg/kg IV, IM or SQ). Bite to antivenom time was <6 h for most canine cases, followed by 6–12, >24 and 12–24 h. Bite to antivenom time was associated with mortality status (P = 0.01) and all cases where antivenom was administered within 6 h survived.

Blood products were administered in 15/88 cases (17.0%); of these, 6 died (40.0%). Most cases received packed red blood cells (pRBCs) only (13/15), after showing marked anaemia (PCV < 20%) combined with the presence of any transfusion trigger, including tachycardia, tachypnoea, hypotension and dull mentation. One case with markedly prolonged coagulation times received fresh frozen plasma (FFP) only and another (a dog with clinical evidence of haemorrhage) received pRBCs, FFP and whole blood to replace red cells and clotting components. Six cases (6.8%) required mechanical ventilation due to exhibiting hypoxaemia (SpO_2_ < 90% or PaO_2_ < 60 mmHg), hypercapnia (PvCO_2_ > 60 mmHg or EtCO_2_ > 60 mmHg), unsustainable breathing effort (as subjectively determined by the treating veterinarian), respiratory arrest or a combination of the above. Patients were ventilated for a median of 13.5 h (4–36 h); of these, 2 died (33.3%). In cases that received antivenom within 6 h, only 1/50 (2.0%) required blood products, although 3/50 (6.0%) required mechanical ventilation.

A mannitol continuous rate infusion (CRI) (0.05–0.1 mg/kg/hr) was administered to 16/88 dogs (18.2%) after developing an AKI or if the treating veterinarian considered the patient to be at high risk of developing an AKI. Four of these dogs were also treated with furosemide. All cases received IVFT (Hartmann's or Plasma‐Lyte 148; Baxter, Australia) and analgesia (methadone SQ or IV, fentanyl CRI and/or a transdermal fentanyl patch [Durogesic®; Janssen‐Cilag, Parkville, Victoria, Australia]). Maropitant (Cerenia®; Zoetis, Australia) and/or gastroprotection (esomeprazole [Nexium® IV Powder for Injection; AstraZeneca, Macquarie Park, NSW, Australia] or omeprazole [Losec®; AstraZeneca]) were given at the discretion of the treating veterinarian. In 51/88 (58.0%) cases, a Foley catheter was placed to monitor urine output and ensure patient comfort, with urine output measured every 4 h.

Of the feline cases, 2/3 received TBAV while the remaining case died before antivenom could be administered.

### Complications

Antivenom reaction occurred in 7/88 dogs (8.0%). In most cases, the reaction was mild and comprised of mild facial swelling (5/7) or generalised erythematous welts (1/7), which resolved either spontaneously or after administration of prednisolone sodium succinate, chlorpheniramine and/or adrenaline. One case became hypotensive shortly after commencing the antivenom injection. The injection was paused and the dog was treated with a fluid bolus, prednisolone sodium succinate, chlorpheniramine and adrenaline. Blood pressure gradually returned to normal and the patient received the remaining antivenom without further complication. Antivenom reactions occurred in patients who received premedication before antivenom administration (4/7) and in those that did not receive premedication (3/7).

AKI developed in 5/88 (5.7%) dogs, of which 4 became anuric following a 0.5–3 day period of oliguria. Within 12 h of developing anuria, 3 dogs were euthanised, whilst the remaining dog went into respiratory arrest and was subsequently euthanised. Serum creatinine concentrations on the day of death were available for 2 cases; one was in IRIS AKI Grade III and the other in Grade IV. Serum K+ on the day of death was not available for any case. The surviving patient was in Grade I (O), which resolved following treatment with furosemide and mannitol. Three/88 (3.4%) dogs developed an arrhythmia. In two cases, this comprised of intermittent ventricular premature complexes that resolved without intervention, while the remaining case developed sustained ventricular tachycardia that was unresponsive to lignocaine therapy; this dog became obtunded, developed concurrent gastrointestinal haemorrhage and was subsequently euthanised. One dog developed evidence of SIRS and MODS, exhibiting an obtunded mentation, pyrexia [temperature 39.8°C], tachypnoea [44 breaths/min], haemorrhagic diarrhoea, generalised subcutaneous oedema and severe abdominal pain. Abdominal ultrasound revealed an oedematous gall bladder wall, pancreatitis and a non‐septic abdominal effusion. Shortly afterwards, the dog developed generalised seizures was euthanised. Clinical haemorrhage occurred in 4/88 (4.5%) dogs. Sites of bleeding included the lung (2 dogs), anterior chamber (2 dogs), gastrointestinal tract (2 dogs), skin (1 dog) and the peritoneal cavity (1 dog). Of the dogs with pulmonary haemorrhage, one dog was treated with blood products (whole blood, FFP and pRBCs) and was mechanically ventilated for 36 h, before it was euthanised after becoming anuric. The other dog was intubated, however, was euthanised shortly afterwards due to the development of concurrent marked hypoglycaemia and hyperlactataemia and concern for the development of SIRS/MODS. Clinical haemorrhage was not the cause of death for the remaining 2 dogs (cases 4 and 6); both received blood products in response to the development of marked anaemia and transfusion triggers; however, they died of other complications.

ARDS developed in one dog that died despite mechanical ventilation. One cat went into cardiopulmonary arrest (CPA) before antivenom could be administered. AKI, cardiac arrhythmias, SIRS, MODS, clinical haemorrhage, or CPA did not occur in any patient where antivenom was administered within 6 h of the bite.

### Outcomes

The median duration of hospitalisation for all cases was 1 day (1–8 days). Of the 88 canine cases, 7 died (8.0%); all after prognosis‐based euthanasia (had treatment continued, the case was highly likely to have died due to continued clinical deterioration, despite a high level of supportive care). One out of three feline cases died (33.3%) after unsuccessful resuscitation following cardiopulmonary arrest. Fatal cases are summarised in Table 4.

**Table 4:** Summary of fatal canine and feline RBBS envenomation cases

Case no.	Date	Signalment	Weight (kg)	Urine result	Bite to TBAV administration (h)	No. vials of TBAV administered	Death after presentation (h)	Cause(s) of death
1	6 January 2019	8‐year‐old, FS Staffordshire bull terrier	13.4	RBBS venom ELISA	>24	1	69	AKI
2	28 May 2017	12‐year‐old, FS, kelpie X	21.0	Black	12–24	1	55	AKI; pulmonary haemorrhage
3	27 November 2015	9‐year‐old, MN, Staffordshire bull terrier	20.9	Black	Unknown	1	24	SIRS and MODS
4	6 January 2015	5‐year‐old MN Staffordshire bull terrier	15.0	Black	> 24	2	84	AKI
5	9 July 2014	8‐year‐old MN Rhodesian ridgeback	40.0	Black	> 24	2	19	AKI; ARDS
6	7 April 2013	13‐year‐old MN Jack Russell terrier	7.4	Black	6–12	1	108	Sustained ventricular tachycardia
7	3 November 2012	2‐year‐old MN ragdoll	4.5	Black	> 24	0	5	CPA
8	22 November 2012	9‐year‐old FS Australian cattle dog	24.0	Black	6–12	1	13	Pulmonary haemorrhage

AKI, acute kidney injury; ARDS, acute respiratory distress syndrome; CPA cardiopulmonary arrest; ELISA, enzyme‐linked immunosorbent assay; FS, female spayed; MODS, multiple organ dysfunction syndrome; MN, male neutered; RBBS, red‐bellied black snake; SIRS, systemic inflammatory response syndrome; SVDK, snake venom detection kit; TBAV, tiger‐brown snake antivenom.

### Multivariable model of risk factors for mortality in dogs

Five variables were initially selected for multivariable modelling using conventional logistic regression; age, respiratory score, PCV and TPP at presentation and bite to antivenom time. A total of 79 cases (73 survivors, 6 deaths) with complete data were available for analysis. On univariate screening, age, a respiratory score ≥ 1, a bite to antivenom time ≥ 12 h, TPP and PCV were selected for inclusion in an initial multivariable logistic regression model (Tables 3 and 4). Age and PCV remained significantly associated with mortality in the final model; older dogs were more likely to die, whereas a higher PCV at presentation was associated with a reduced risk of death (Table 5). Pigmenturia was included in a further multivariable model based on these 79 dogs, using Firth's method,^19^, ^20^ with age and pigmenturia remaining in the final model (Table 6).

**Table 5:** Initial multivariable and final multivariable associations between potential risk factors and death for 79 canine RBBS envenomation cases with complete data

Initial multivariable model			Final multivariable model	
Predictor variable	OR (95% CI)	P value	OR (95% CI)	P value
Age (months)	1.03 (1.00–1.07)	0.04	1.03 (1.00–1.06)	0.03
Respiratory score				
0				
≥1	3.27 (0.27–39.96)	0.35		
Bite to AV time				
<12 h				
≥12 h	2.65 (0.24–29.25)	0.43		
PCV (%)	0.94 (0.87–1.02	0.16	0.92 (0.85–0.98)	0.01
TPP (g/L)	1.06 (0.97–1.17)	0.19		

CI, confidence interval; OR, odds ratio.

**Table 6:** Initial and final multivariable logistic regression models of risk factors for death in 79 canine RBBS envenomation cases (6 died) with complete data, using the Firth method of penalized maximum likelihoods to adjust for non‐convergence due to quasi‐complete separation in pigmenturia

Predictor variable	Initial multivariable model OR (95% CI)	P value	Final multivariable model OR (95% CI)	P value
Age (months)	1.02 (1.00–1.05)	0.05	1.03 (1.00–1.05	0.02
Pigmenturia	5.23 (0.31–88.47)	0.25	20.65 (1.09–391.96)	0.04
Respiratory score				
0				
≥1	2.48 (0.39–15.82)	0.34		
Bite to AV time				
<12 h				
≥12 h	1.20 (0.17–8.36)	0.86		
Initial PCV (%)	0.97 (0.90–1.05)	0.48		
Initial TPP (%)	1.05 (0.97–1.13)	0.21		

CI, confidence interval; OR = odds ratio.

Another conventional logistic regression analysis, including ACT as well as the previous risk factors, was then performed. Around 56 cases (51 survivors, 5 deaths) with complete data were available for analysis and ACT was positively associated with death on both univariate (*P* = 0.01) and multivariate analysis (*P* = 0.02) (Table 7). When pigmenturia was included and Firth's method used, age and TPP remained in the final model's risk factors for death (Table 8). Further models incorporating the variables already assessed and additionally serum K+ and iCa, were explored; however, complete data was available only for 42 dogs with 4 deaths; this sparse data in the subsets of available data precluded the development of reliable models.

**Table 7:** Initial multivariable and final multivariable associations between potential risk factors and death for 56 canine RBBS envenomation cases with complete data

Univariate logistic regression			Final multivariable model	
Predictor variable	OR (95% CI)	P value	OR (95% CI)	P value
Age (months)	1.10 (0.97–1.25)	0.13	1.09 (1.01–1.17)	0.03
Respiratory score				
0				
≥1	0.88 (0.01–69.90)	0.95		
Bite to AV time				
<12 h				
≥12 h	0.23 (<0.001–56.51)	0.60		
PCV (%)	0.98 (0.88–1.09)	0.68		
TPP (g/L)	1.13 (0.87–1.48)	0.36		
ACT (SNDs)	1.71 (0.88–3.30)	0.13	1.88 (1.12–3.16)	0.02

CI, confidence interval; OR, odds ratio.

**Table 8:** Initial and final multivariable models for the association between potential risk factors and death for 56 canine RBBS envenomation cases (5 died) with complete data, using the Firth method of penalized maximum likelihoods to adjust for non‐convergence due to quasi‐complete separation in pigmenturia

Univariate logistic regression			Final multivariable model	
Predictor variable	OR (95% CI)	P value	OR (95% CI)	P value
Age (months)	1.02 (1.0–1.04	0.07	1.03 (1.0–1.06)	0.05
Pigmenturia	1.14 (0.12–10.91)	0.91		
Respiratory score				
0				
≥1	0.67 (0.10–4.48)	0.68		
Bite to AV time				
<12 h				
≥12 h	1.82 (0.18–18.50)	0.61		
PCV (%)	0.96 (0.87–1.03)	0.23		
TPP (g/L)	1.05 (0.96–1.15)	0.29	1.11 (1.02–1.22)	0.02
ACT (SNDs)	1.10 (0.89–1.37)	0.39		

## Discussion

This study confirms and extends our understanding of the RBBS toxidrome in dogs and cats, identifying six risk factors for mortality on univariate analysis that could guide treatment and prognosis in future cases. Compared to survivors, dogs that died were older, exhibited pigmenturia, received antivenom later and had a higher TPP and ACT and lower PCV at presentation.

Delayed antivenom administration presumably allows circulating myotoxins and hemolysins to exert their effect, leading to rhabdomyolysis and haemolysis manifesting as anaemia, hypocalcaemia (due to calcium entering damaged myocytes and calcification of necrotic muscle) and elevated TPP.^21^ This finding is supported by findings in people that antivenom given ≤6 h after the bite prevents the development of myotoxicity and promotes rapid normalisation of coagulopathy.^22^, ^23^ Pigmenturia is presumably associated with both myoglobinuria and haemoglobinuria, consequences of delayed antivenom administration, haemolysis and myolysis. Of note, all dogs without pigmenturia survived; thus, the absence of pigmenturia at presentation is a good prognostic sign. Conversely, all dogs that died had pigmenturia, as did nearly 40% of the survivors, so pigmenturia does not rule out survival.

In addition, these patients are at risk of myohaemoglobinuric AKI. The pathophysiology for this phenomenon is yet to be fully elucidated but is thought to involve a combination of renal tubular cytotoxicity (caused by excessive glomerular filtration of haemoproteins which are taken up by tubular cells where they generate reactive oxygen species and mediate pro‐inflammatory effects), renal vasoconstriction (due to activation of the renin‐angiotensin‐aldosterone system secondary to fluid sequestration within damaged muscle, as well as an imbalance between vasoconstrictors and vasodilator products that regulate renal blood flow) and intratubular casts formation (from the reaction of haemoproteins with Tamm‐Horsfall tubular protein).^24^, ^25^, ^26^

AKI was the most common cause of prognosis‐based euthanasia in our study population; the question arises of how we prevent the continued haemolysis that occurs in some cases despite TBAV administration and the lack of detectable free RBBS venom antigen following this.^9^ Administering multiple vials of TBAV remains a contentious issue, with proponents advocating that TBAV may be inefficient at neutralising black snake hemolysins and that free venom antigen ELISA measurements in the presence of high concentrations of antivenom may be unreliable, while critics argue that the haemolytic pathology may be rapid and largely irreversible, rendering delayed antivenom administration largely useless.^3^, ^9^ These hypotheses do not, however, account for prior observations that dogs receiving prompt antivenom treatment have an excellent prognosis and generally do not show evidence of continued haemolysis.^2^, ^3^ Further work investigating the mechanism of RBBS envenomation‐associated haemolysis is warranted. From clinical experience, the authors recommend that in cases with the rapid development of intravascular haemolysis, rapid decrease in PCV and/or severe myoglobinaemia/uria, more than one vial of TBAV may be indicated either at presentation or on subsequent days if haemolysis and progressive anaemia continue. The development of oliguric/anuric AKI in veterinary patients is often fatal. Renal replacement therapies, using haemoprotein‐permeable membranes, would be the ideal treatment for RBBS envenomation‐induced AKI, although limited availability in veterinary hospitals and high cost makes this a prohibitive option in most cases. The authors are not aware of any cases that have been treated with renal replacement therapy. Other therapeutic options that could be explored include urine alkalinization (which theoretically will inhibit precipitation of haemoproteins with Tamm‐Horsfall protein) and plasmapheresis, although there is no evidence of clinical benefit for either in humans with rhabdomyolysis‐induced AKI.^21^, ^25^, ^26^

Although prolonged ACT at presentation was associated with death, coagulopathy remained subclinical in most cases, with only four cases in our study population showing active haemorrhage. As such, ACT may be used as a marker of envenomation severity though the coagulopathy induced by RBBS‐envenomation is rarely of clinical concern. Older age, the final risk factor for mortality we identified, was an unexpected finding. We speculate that older dogs may be more susceptible to complications arising from RBBS envenomation, increasing their risk of death. Alternatively, older age may also influence a clinician's or owner's decision to consider euthanasia; however, this is less likely to play a role given that all euthanised patients had a poor prognosis with continued treatment regardless of age.

It is interesting to note that the fatal complications documented in these cases do not seem to occur in people where the RBBS toxidrome is limited to a localised bite‐site reaction, subclinical coagulopathy and occasional myotoxicity.^23^, ^27^ Reasons for this are unclear but may include physiological and/or behavioural differences between species (e.g. dogs may fight back and get bitten multiple times, whereas humans may retreat immediately following the first bite). The greater bodyweight of humans may be less likely to play a role, given that bodyweight was not associated with death in our study.

Although the canine toxidrome of haemolysis, myopathy, pigmenturia, bite site oedema and mild coagulopathy has been well described previously,^2^, ^3^ our comparatively large study population allows us to define additional observations which we hope will be helpful to veterinarians practising in areas where the RBBS is endemic. Importantly, the absence of clinical signs, haemolysis or prolonged ACT at presentation does not rule out envenomation and a urine SVDK should be performed or prophylactic TBAV given in cases where there is an owner history of dog‐snake interaction. Antivenom reactions are usually mild and infrequently occur regardless of whether the patient receives pre‐medication before administration, which is consistent with previous findings.^28^ As such, we recommend against premedication prior to the administration of antivenom, rather only using prednisolone sodium succinate, chlorpheniramine and/or adrenaline to treat a hypersensitivity reaction if it occurs. Owners may be counselled that dogs presenting within 6 h of envenomation have an excellent prognosis with TBAV administration and supportive care, while dogs presenting later may experience potentially fatal complications such as AKI, SIRS, MODS, ARDS and haemorrhage. Finally, RBBS envenomation appears to be relatively rare in cats. The three feline cases reported here seem consistent with the canine toxidrome, although interestingly neurotoxic and myotoxic effects seem to predominate with no evidence of haemolysis observed. Perhaps the disparity between species may reflect behavioural differences placing dogs at higher risk of snake exposure, although this does not seem to be the case with Eastern brown snake (*Pseudonaja textilis*) envenomation with dogs and cats seen at similar frequencies.^14^ More reports of feline RBBS envenomation are required before the toxidrome can be accurately described in cats.

The main limitations of this study stem from its retrospective nature. Firstly, a complete dataset for all variables was unavailable for every case. This limited our sample size for logistic regression analysis; consequently, our conclusions should be considered speculative and we recommend further studies involving a much larger sample size be performed to validate our findings. In addition, specific clinical signs were assumed to be absent if they were not documented as present in the medical record and as such, some clinical signs may have been under‐reported. The accuracy of TPP measurements obtained from refractometry readings may have been affected by haemoglobinaemia and myoglobinaemia; however, the effect is probably minor.^29^, ^30^ Similarly, haemoglobinaemia and myoglobinaemia may have affected the accuracy of some serum biochemistry readings obtained (e.g. bilirubin, AST, CK).^31^ Finally, these cases were all from a relatively small geographic area and it is unclear whether or not our findings are generalisable to dog and cat RBBS envenomation cases from other geographic regions.

Although RBBS envenomation carries a generally excellent prognosis with appropriate treatment, old dogs, pigmenturia, delayed antivenom administration and a higher TPP and ACT and lower PCV at presentation are risk factors for mortality. AKI secondary to rhabdomyolysis and haemolysis is the most common complication of treatment and is often fatal despite TBAV administration and intensive supportive care. SIRS, MODS, ARDS and haemorrhage may also occur. In high‐risk cases, advanced treatment modalities such as renal replacement therapies and plasmapheresis should be considered for optimal outcomes, although prompt TBAV administration remains the mainstay of successful treatment.

## Conflicts of interest and sources of funding

The Padula® TBAV used in this study were manufactured by Dr Andrew Padula of Padula Serums Pty Ltd, Australia.
